# Harnessing DNA Synthesis to Develop Rapid Responses to Emerging and Pandemic Pathogens

**DOI:** 10.4061/2011/765763

**Published:** 2011-03-16

**Authors:** Lisa M. Runco, J. Robert Coleman

**Affiliations:** ^1^New York Institute of Technology (NYIT), Department of Life Sciences, Old Westbury, NY 11568-8000, USA; ^2^VitaCode Biotechnology LLC, Research and Development, Box 145, Blauvelt, NY 10913-0145, USA

## Abstract

Given the interconnected nature of our world today, emerging pathogens and pandemic outbreaks are an ever-growing threat to the health and economic stability of the global community. This is evident by the recent 2009 Influenza A (H1N1) pandemic, the SARS outbreak, as well as the ever-present threat of global bioterrorism. Fortunately, the biomedical community has been able to rapidly generate sequence data so these pathogens can be readily identified. To date, however, the utilization of this sequence data to rapidly produce relevant experimental results or actionable treatments is lagging in spite of obtained sequence data. Thus, a pathogenic threat that has emerged and/or developed into a pandemic can be rapidly identified; however, translating this identification into a targeted therapeutic or treatment that is rapidly available has not yet materialized. This commentary suggests that the growing technology of DNA synthesis should be fully implemented as a means to rapidly generate *in vivo* data and possibly actionable therapeutics soon after sequence data becomes available.

## 1. Pandemic Viral Outbreaks

Today, the ability to determine if an isolated viral outbreak could develop into a pandemic has been facilitated by efficient PCR and sequencing techniques to quickly identify and characterize the pathogen [1]. The prompt generation of sequence data from infected individuals has allowed for the identification of these emergent pathogens and for the Center for Disease Control (CDC) or World Health Organization (WHO) to determine if these emergent pathogens pose a pandemic threat. This determination is based on the early rate of infection, sequence data similarity, and virulence factor molecular markers [2, 3]. Take for example the recent pandemic of the 2009 H1N1 Influenza A virus (2009 H1N1) which was identified in Mexico and rapidly spread to other countries [4]. Sample isolation and sequencing provided for immediate analysis of the sequence data and determination of origin, strain, and genomic characteristics of the virus [5]. Thus, health agencies could hypothesize that indeed it was a threat to the global community given its antigenic novelty [6]. The CDC has estimated that the H1N1 pandemic infected between 47 to 81 million individuals [7]. The majority of individuals infected with 2009 H1N1 experienced mild disease symptoms, yet it was estimated that the disease accounted for nearly 9,820 deaths in the United States (US) alone [7]. Influenza virus is a continual threat as the cause of a pandemic outbreak given the ability of the virus to reassort via the phenomenon of antigenic shift. 

Antigenic shift is the result of a host being infected with two or three different influenza strains. While replicating in the host, these viruses exchange segments. This genome fragment-swapping could yield a virus with an antigenic profile that is completely novel to the human-host population, allowing for rapid spread [8]. This process of antigenic shift is hypothesized to be the generating event for the 2009 H1N1 virus. Amazingly, the 2009 H1N1 virus was the result of multiple rounds of reassortment that actually combined portions of avian, swine and human influenza viruses, ultimately yielding the virus strain which spread rapidly across the globe [9]. By combining segments from three progenitor strains, the resulting 2009 H1N1 virus was highly variable, allowing for rapid transmission among immunologically naïve human-hosts [10]. The 2009 H1N1 pandemic was not the only example of influenza spreading across the globe. Other outbreaks include the mild, with regards to morbidity, but wide-spread 1964-1965 Hong Kong influenza, as well as the infamous 1918 Spanish influenza pandemic, which was severe and responsible for an estimated 50 to 80 million deaths [11]. 

Aside from the influenza pandemics, an entirely unrelated coronavirus was responsible for a significant emergent outbreak in 2002 that spread to numerous locations across the globe [12]. This was the well-publicized SARS virus which initially began in the Guangdong province of China and spread globally to 37 countries [13]. Initially, the exact viral cause of SARS was unknown until the implementation of the virus chip by Wang et al. allowed for its identification as a coronavirus [14, 15]. This virus was estimated to be the causative agent in the morbidity of 8,000 individuals, with a resulting mortality rate of 10% [12]. Despite having extremely different genetic compositions (i.e., influenza is a (−) RNA virus and coronaviruses are (+) RNA viruses), they share the characteristic of cross-transmission. These viruses are capable of infecting a range of mammalian and avian hosts. Infection of humans usually manifests as a severe upper respiratory disease [16]. 

Both of these example viruses, 2009 H1N1 and SARS, were identified and characterized based on sequence data, but targeted, rapid treatments were not readily produced using this sequence information. For example, the main treatment and control measures implemented for SARS were simply isolation of infected individuals. This included quarantining infected individuals, quarantining any patients presenting an upper-respiratory disease in hospitals, limiting travel, avoidance of public places, and implementing strict hygiene practices in hospitals [17, 18]. Not to disparage good hygiene as an effective means to combat infection, but given current medical advancements a more targeted treatment should be sought to combat these outbreaks. 

The first line of treatment of the 2009 H1N1 was also rather low technology and uninspiring. Administration of the currently available antivirals Oseltamivir (Tamiflu) and Zanamivir [10] and simple measures such as school-closures and quarantine [19] were implemented. Vaccine production against 2009 H1N1 was slow, resulting in a vaccine available for public consumption in the late Fall of 2009, approximately 6 months after the first diagnosed case in Mexico. By the time the vaccine was being administered, the virus had spread globally, infecting an estimated 6 million people in the US alone [5]. To compound this lack of response, there were persistent vaccine shortages which highlight the inefficiency in the currently available treatment repertoire [7]. Fortunately, this virus was rather mild; however, these shortages, in the case of a pandemic, would lead to a higher mortality rate, as well as civil unrest and increased panic.

When analyzing the monthly sequence data from patients infected with 2009 H1N1, by May 2010, 54 Oseltamivir-resistant Influenza A virus (2009 H1N1) isolates had been identified by the CDC, demonstrating that antiviral resistance rapidly emerged [20]. Resistance is identified by an amino acid substitution of H275Y in the neuraminidase gene [21]. Therefore, the widespread use of antivirals as a rapid treatment for a pandemic outbreak may prove to be a stopgap that is not completely effective. Initially antivirals would slow the progression of infection but once resistant strains emerge, these treatments would prove ineffective. The first isolated Oseltamivir-resistant strain of the 2009 H1N1 in the US was detected the week of August 2, 2009, four months after the first case was reported in Mexico [22]. Thus, current drug-based therapies may prove ineffective especially if resistant strains recombine with a more severe virus. 

We propose the harnessing of rapid DNA synthesis to develop targeted treatments based on emerging sequences as an approach for swift clearance of newly emergent pandemic strains [23]. Rapid DNA synthesis may allow for an adaptable response and treatment that can change along with the mutating pathogen. Granted regulatory bodies could prove to be a hindrance in the quick development and dissemination of a novel therapeutic agent based on synthetic DNA; however, these policies may change if or when we are faced with a true pandemic threat such as the 1918 flu, which resulted in 50 to 80 million of deaths.

## 2. Emerging Pathogens

Aside from the known pandemic threats outlined above, other emerging pathogens also threaten the local and, or global community should they escape their current niché and begin to infect large human populations. Previously, an emergent outbreak would have been limited to a single geographic location, but given the rise of global travel these pathogens could impact the health of the global community [24]. Examples of these emergent pathogens include outbreaks from food-borne pathogens [25], nosocomial outbreaks (i.e., SARS in Toronto which originated in China) [26], and lastly bioterrorist agents such as smallpox, which has been eradicated from the general population [27]. Currently, the CDC has developed an entire organization designated “The Division of Emerging Infections and Surveillance Services” (DEISS), dedicated to monitoring emerging pathogens as they appear, hoping to identify causative agents and contain any transmission. Aside from the CDC, entire research institutions have been established to focus on emergent pathogens and their impact on global health (e.g., Global Health & Emerging Pathogens Institute at Mount Sinai School of Medicine and The Emerging Pathogen Institute at The University of Florida). Since some of these emergent infectious outbreaks come from zoonotic sources that have made a “jump” to the human host, the effective treatment options for humans are unknown and/or poorly characterized. However, given that sequence data could be quickly generated using known procedures, new treatments that harness rapid *de novo* DNA synthesis may be the only means to target and specifically combat emergent outbreaks as they arise.

## 3. Economic Impact: Halting Pandemics Rapidly in order to Prevent Economic Hardship

Aside from the obvious widespread morbidity and mortality should a pandemic occur, there would be additional economic fall out that would also have a detrimental impact on society. The cost to treat the patients of a pandemic would be high. Using the example of SARS, which only impacted a few individuals but had an extremely exuberant political and medical response [28], it was estimated that the cost of the outbreak ranged from $3 to $10 million per patient [29, 30]. This is a significant sum when realizing the small quantity of individuals infected during the SARS outbreak. With regards to a pandemic influenza outbreak, the health care costs alone are estimated to range from USD $71.3 to $166.5 billion, excluding its economic impact on business [31]. When analyzing a pandemic outbreak one must view the impact on the economy as a whole. One study presented at a forum conducted by the Australian Center for Economic Research on Health performed an in-depth analysis of a pandemic influenza and its alteration of global economic output [32]. This study by Sidorenko et al. divided a potential pandemic into four scenarios: mild, moderate, severe, and ultra, with each based on the mortality rate of a possible pandemic and their resulting economic consequences [32]. These divisions are necessary when analyzing a hypothetical pandemic because the potential economic impact varies depending on the severity of the virus. The estimated death total in the United States for these four scenarios would be 20, 201, 1,000.9, and 2,018.9 (in thousands), respectively. These estimates are multiplied by 2x to 10x if estimating mortality in developing countries. Examining the estimates for the economic impact in the US alone, the estimated reductions in gross domestic product (GDP) for these four categories are −0.58%, −1.38%, −3.00%, and −5.50%, respectively [32]. Globally, a mild pandemic like the current 2009 H1N1 virus would cost USD $330 billion or 0.8% GDP. An ultra pandemic, akin to the 1918 Influenza A virus, is estimated to have a substantial global economic impact with a cost of USD $4.4 trillion or 12.6% GDP [32]. The astounding economic cost is a result of decreased productivity, changes in behavior (i.e., avoidance of public places, going to work, etc.), adjusted interest rates, inflation, and shear shock to the public and society. Specifically, in an ultra pandemic the study estimated an additional 2.22% rise in inflation in the United States, a 9.6% decrease in exports, and −101 basis point reduction in short-term interest rates [32]. Given that the US is a first world society with a modern health care system capable of combating a potential pandemic, these totals are even more pronounced when looking at projections for the developing world. In sum, the developing world death rates and GDP reductions would be far greater than the US. The economic impact in the developing world would be substantial due to the shock from human loss of life as well as the flow of capital from emerging markets to “safe havens” such as the US and the EU [32].

While the ultra pandemic is an extremely rare event one never hopes to encounter, these astounding numbers underscore the need for rapid targeted treatments. The emergence of antiviral resistant strains indicates the need for the harnessing of DNA synthesis as a means to develop target therapeutics to replace current antivirals. These *de novo* DNA-based therapeutics could be customized to sequences as they are isolated in the field, allowing for adaptability of treatment.

## 4. Synthetic Biology: The Emergence of DNA Synthesis and Its Promise for Rapidly Available Treatments

**Figure 1:**
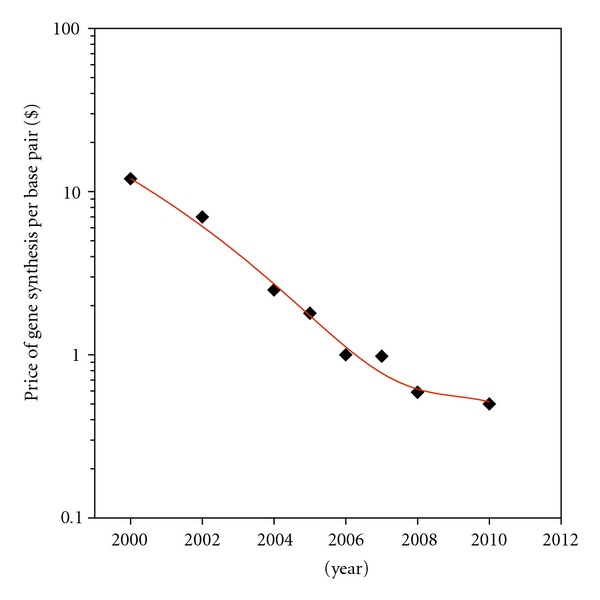
The decrease of large-scale *de novo* DNA synthesis costs. This graph depicts the decrease in the price per base of large fragment DNA synthesis [23]. As the price decreases, the greater is the applicability of large-scale DNA synthesis as a functional means to combat emerging and pandemic pathogens. The price indicated is defined in US Dollars.

The new field of synthetic biology has emerged and has illuminated the possibility of rapidly generating therapeutic agents against emerging threats as they arise and threaten communities or the globe. Specifically, the scientific community's ability to synthesize *de novo*, without a natural template, genetic material in the form of DNA has enabled synthetic biology [23]. Now the door is open to a vast arena of new experiments and treatments because the ability to customize and rapidly generate genetic material is a reality. For example, pathogens with synthetically designed genomes that are attenuated could serve as vaccines [33, 34]. In essence, a researcher can now convert digital sequence data into biologically relevant genetic material in relatively short order. Currently, the cost of synthesis is approximately 39 cents per base synthesized [35]; however, given the rapid improvement of this technology, the price per base is estimated to decrease throughout the decade (Figure 1) [23]. There are now 39 companies that offer complete gene synthesis, a remarkable number, and a resource that should be utilized by research laboratories [36]. On average when placing a nonrushed order, some biotechnology companies take 7 to 10 days to yield 1 kb of DNA. However, certain companies (such as Blue Herron Biotechnology and GENEART) have advertised that any sequence relating to the H1N1 virus will be rapidly generated within 2 to 4 days. A specific press release by GENEART stated the company rapidly generated genes (one over 1,800 base pairs) of viral coat proteins for H1N1 in just 3 days [37]. The shear speed at which this DNA can be generated is astounding. It indicates that it is possible to have a targeted treatment that utilizes this rapidly generated DNA ready for an emergent threat in a short period of time. Therefore, new methods to combat infectious agents must be nucleic acid-based and thus technologies based on nucleic acid should be pursued.

It will also be cost effective to use synthetic DNA-based therapeutics given the previous method of developing small molecule inhibitors of microbes. Previously these molecules have been developed through screen of chemical libraries for effectiveness against a certain pathogen [38]. This is a brute force approach to drug development that could be a waste of resources. Given the ability to synthesize genetic material, a more targeted and specific approach should be taken. This means no longer are drugs developed by trial and error, but rather in a thoughtful and targeted manner.

## 5. siRNA Delivery As an Antiviral

One application of DNA synthesis would be the generation of platform for the delivery of RNAi to inhibit an infecting virus. These specific RNAs could be synthesized rapidly for the target pathogen and thus be used as a targeted treatment approach. This method has successfully controlled plant virus infectivity [39]. In addition, *in vivo* work has shown effectiveness in mammals [40]. Specifically Hepatitis B replication was controlled in infected mice [40] and numerous studies have shown that siRNAs are able to slow Herpes replication in neurons and target tissue [41]. The major limitation is the delivery of these RNA molecules, which still requires further investigation. However, once the delivery hurdle has been crossed, the advent of *de novo* nucleic acid synthesis should allow for their rapid, targeted production. In order for inhibitor RNA molecules to function, they require high sequence similarity to their target. Given that viruses are continually mutating, a single RNA molecule that would function as a treatment is unlikely; however, being able to quickly generate genetic material now allows for the construction of inhibitory RNAs as their intended target mutates in the field.

## 6. Rapid Data on Emergent and Mutating Strains

Another advantage of DNA synthesis is its applicability to the study of pathogens *in vitro*. Previously, biomedical laboratories relied on PCR and standard mutagenesis to study viral variants that arose via mutation. These studies were focused on the impact these mutations had either on viral growth [42, 43], *in vivo* pathogenesis [44], or the ability of the mutations to confer drug resistance [45]. Now looking through the lens of DNA synthesis, this process was extremely laborious given the small number of mutations that could be studied. Granted important findings and thorough work resulted from using these methods; however, DNA synthesis now affords us the ability to generate a vast number of variations of a sequence, which can be screened for biological relevance. For example, field isolates with sequence variation could be generated quickly for direct study in the laboratory, allowing for rapid data on these emerging variants. Thus, nucleotide changes seen in almost real-time from clinical isolates could be studied in the laboratory setting.

## 7. Targeted Peptide or Recombinant Protein Vaccines

The rapid generation of sequence data from emerging pathogens may also serve to enhance the prospects of peptide- or recombinant protein- (RP) based vaccines. One can readily foresee the applicability of harnessing rapidly generated sequence data as a means of producing a targeted peptide or RP vaccine against an emergent threat. The antigenic or coding sequences attained from the sequence of an emergent pathogen can be swiftly harnessed by DNA synthesis and translated into an immediately available peptide vaccine. However, the current drawbacks of low immunogenicity of peptide vaccines, weak adjuvants, and/or the lack of optimal carrier molecules will require further enhancement until the approach of peptide- or RP-based vaccines as anti-infectives is completely viable [46]. There have been recent advances using the peptides, for example, HP0245, as a protective peptide vaccine capable of protecting animals against the emerging zoonotic pathogen *Streptococcus suis* serotype 2 [47], as well as peptide-induced immunity to *Mycobacterium tuberculosis* in humans [48]. Additionally, the utilization of recombinant viral or bacterial vectored vaccines, such as adenovirus vector systems, could be implemented as a quick response vaccine to an emergent microbe. There has been a recent effort to utilize replication-deficient adenovirus vectors as possible HIV vaccines with some success in providing enhanced immunity to HIV in nonhuman primates [49]. The advantage of using viral vectors is that they induce a strong CD4^+^ and CD8^+^ T-cell response to your antigen of interest [50]. There are still drawbacks to viral vector vaccines that require resolution, such as the possibility of preexisting immunity to the vector, in turn preventing immunization as well as a weak humoral response to the “vaccinating” transgenes, which may be required for protection [51]. Thus, like the peptide vaccine strategy, these too need further optimization before viral vectors are a readily available system capable of rapid use against an emergent pathogen.

## 8. Customized, Attenuated Vaccines

Lastly, one of the most effective treatments for infectious diseases available today is the live-attenuated vaccination. It has successfully decreased morbidity and mortality across the globe, as well as eradicated some human pathogens. Previously, live-attenuated vaccines have been developed by serial passages of viruses in nonhuman cells, rendering them less pathogenic upon return to the human-host [52]. This strategy is an approach that relies on random mutations, rather than user-directed attenuation. Recently, synthetic biologists have been recoding viral pathogens (poliovirus and Influenza A virus) with synonymous codon-pairs as a means of attenuation [33, 34]. In these studies, the codon-pair bias of poliovirus and Influenza A virus were altered at the genome level, such that the viral genome's translation efficiency was down-modulated via synthetic alteration. Large fragments of their genomes were “recoded” with under-represented (i.e., “slow”) codon-pairs. This recoding maintained the amino acid identity at the protein level, yet incorporated over 400 nucleotide level synonymous mutations, which ultimately altered the genome's translation rate. The ability to alter genetic material on a macro-scale was enabled by combining the functionality of gene-design computer software with large-scale DNA synthesis [23]. These synthetically modified viruses were attenuated in animal models. According to the New England Journal of Medicine, this method holds promise as a platform for construction of live-attenuated vaccines [53]. Since this method relies on DNA synthesis and universally applicable computer software, this technology has the potential for application to yield live-attenuated vaccines against viral threats as they emerge [35].

## 9. Conclusions

In sum, we have recently seen, when considering the 2009 H1N1 pandemic influenza, that the threat of a global pandemic is real and that fortunately this most recent outbreak was by a virus with reduced pathogenicity. However, this has heightened the biomedical communities' awareness that treatments and containment strategies for pandemic outbreaks must be further improved and developed. These include responses that are rapid in nature, that are targeted, and that contain the spread of infection. This commentary suggests that the implementation of DNA synthesis-based therapeutics and strategies deserve serious consideration as treatments. DNA synthesis provides targeted adaptability, that is, targeted treatments that could be rapidly generated and adapted as the viral outbreak mutates.
